# A multi-centre, randomized, controlled trial on coaching and telemonitoring in patients with cystic fibrosis: conneCT CF

**DOI:** 10.1186/s12890-021-01500-y

**Published:** 2021-04-21

**Authors:** Stephanie Thee, Mirjam Stahl, Rainald Fischer, Sivagurunathan Sutharsan, Manfred Ballmann, Axel Müller, Daniel Lorenz, Dominika Urbanski-Rini, Franziska Püschner, Volker Eric Amelung, Carola Fuchs, Marcus Alexander Mall

**Affiliations:** 1 Department of Pediatric Respiratory Medicine, Immunology and Critical Care Medicine, Division of Cystic Fibrosis, Charité – Universitätsmedizin Berlin, Berlin, Germany; 2 German Centre for Lung Research (DZL), Associated Partner, Berlin, Germany; 3 Cystic Fibrosis Centre Munich-West, Munich, Germany; 4 Department of Pulmonary Medicine, Division of Cystic Fibrosis, University Medicine Essen -Ruhrlandklinik, University of Duisburg-Essen, Essen, Germany; 5 Department of Pediatrics, University Medicine Rostock, Rostock, Germany; 6 m.Doc GmbH, Köln, Germany; 7 Thieme TeleCare Ltd., Stuttgart, Germany; 8 Private Institute for Applied Health Services Research (Inav) GmbH, Berlin, Germany; 9 PARI Medical Holding GmbH, Starnberg, Germany; 10 Berlin Institute of Health (BIH), Berlin, Germany

**Keywords:** Cystic fibrosis, Telemedicine, Adherence, Home spirometry

## Abstract

**Background:**

The extend of lung disease remains the most important prognostic factor for survival in patients with cystic fibrosis (CF), and lack of adherence is the main reason for treatment failure. Early detection of deterioration in lung function and optimising adherence are therefore crucial in CF care. We implement a randomized controlled trial to evaluate efficacy of telemonitoring of adherence, lung function, and health condition in combination with behavior change interventions using innovative digital technologies.

**Methods:**

This is a multi-centre, randomized, controlled, non-blinded trial aiming to include 402 patients ≥ 12 years-of-age with CF. A standard-of-care arm is compared to an arm receiving objective, continuous monitoring of adherence to inhalation therapies, weekly home spirometry using electronic devices with data transmission to patients and caring physicians combined with video-conferencing, a self-management app and professional telephone coaching. The duration of the intervention phase is 18 months. The primary endpoint is time to the first protocol-defined pulmonary exacerbation. Secondary outcome measures include number of and time between pulmonary exacerbations, adherence to inhalation therapy, changes in forced expiratory volume in 1 s from baseline, number of hospital admissions, and changes in health-related quality of life. CF-associated medical treatment and care, and health care related costs will be assessed by explorative analysis in both arms.

**Discussion:**

This study offers the opportunity to evaluate the effect of adherence interventions using telemedicine capable devices on adherence and lung health, possibly paving the way for implementation of telemedicine in routine care for patients with CF.

*Trial registration*: This study has been registered with the German Clinical Trials Register (Identifier: DRKS00024642, date of registration 01 Mar 2021, URL: https://www.drks.de/drks_web/navigate.do?navigationId=trial.HTML&TRIAL_ID=DRKS00024642).

**Supplementary Information:**

The online version contains supplementary material available at 10.1186/s12890-021-01500-y.

## Background

Cystic fibrosis (CF) is an inherited multi-organ disorder that causes damage mainly to the lungs, but also to organs of the digestive system such as the pancreas and liver (1, 2). CF is caused by mutations in the cystic fibrosis transmembrane conductance regulator (*CFTR*)-gene leading to abnormal function of the CFTR anion channel in the epithelium of different organs. The recent development of highly effective CFTR-directed therapeutics is currently transforming the care of patients with CF leading to an increase in lung function, an improvement in quality of life and a decrease in pulmonary exacerbations (3–5). However, the extent of the lung disease with lung function as a surrogate marker is the most important prognostic factor for survival (6, 7). Irreversible lung damage, e.g. bronchiectasis, has often already occurred in pediatric and adult patients by initiating of the new CFTR-directed therapies (8). Therefore, inhalation therapies with antibiotic and mucolytic agents remain a cornerstone of symptomatic therapy (1, 9, 10). These therapies are incredibly time-consuming and adult patients with CF usually spend at least 1.8 h per day performing an average of seven inhalations (11). In the face of this immense treatment burden, lack of treatment adherence was identified as the leading cause for treatment failure (12). Compared to electronic monitoring, which shows adherence to only 30–50% of all prescribed treatments, self- and clinician-reported adherence overestimate adherence substantially (11, 13–15). In the current standard of care, the CF physician has no objective information on the patient’s adherence, state of health and quality of life between the scheduled visits. Readily available information on adherence and lung function would allow for greater patient self-management of the disease and also for early intervention and adjustment of therapies by the CF physician, potentially preventing pulmonary exacerbations and slowing lung disease progression.

The role of digital technologies in supporting patients with CF in managing their complex therapies is being more and more acknowledged (16). Recently developed data-tracking nebulizers enabling patients to inhale all standard medications allow for accurate monitoring of inhalation therapies. Home monitoring strategies, including spirometry and symptom reporting, have been used for early detection of pulmonary exacerbations with conflicting results (17–20). Patients with CF are often cared for in specialized CF clinics. Regular follow-up visits delivered by trained, multidisciplinary teams have led to improvements in CF care and patient outcomes, but this centralization means longer travel distances for patients, resulting in more missed school and work. Especially in between routine visits, video-conferencing appears to be a promising option for delivering health care while reducing travel time, but has only been assessed in small studies (16).

This multi-centre trial aims to evaluate telemedicine’s effect including telemonitoring of inhalation therapy and lung function, a self-management app, video-conferencing and telephone coaching on adherence to treatment, lung function, and pulmonary exacerbations in patients with CF. The aforementioned digital technologies, combined with individualized phone coaching by psychologists as an intervention for behaviour change, are applied to achieve this goal. We hypothesize that this intervention leads to improved adherence, a prolonged time between pulmonary exacerbations, improved lung function and higher quality of life than patients receiving routine care.

## Methods/design

### General study protocol

**Fig. 1:**
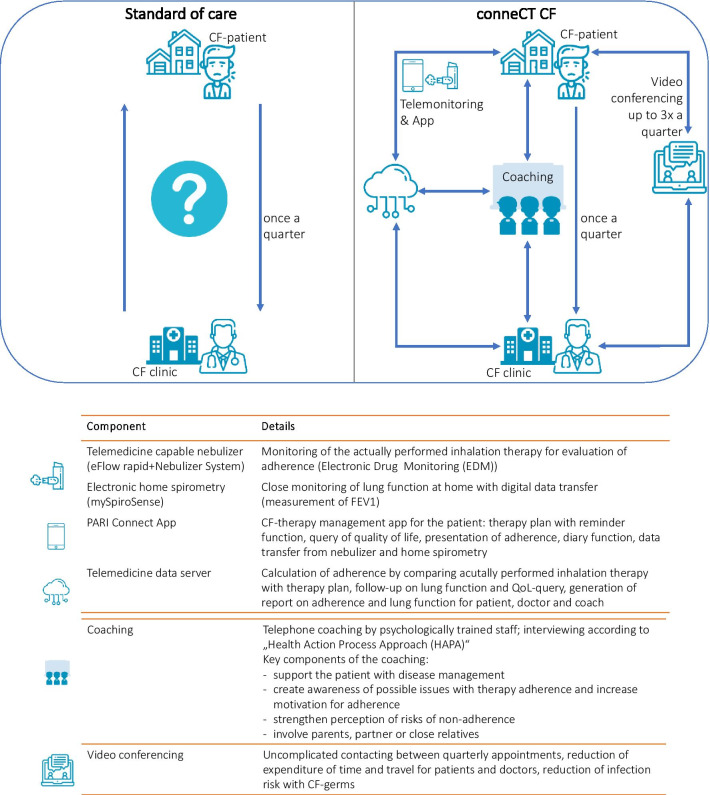
Standard of care for patients with CF compared to the intervention group in connect CF. A standard-of-care arm is compared to an intervention arm using objective, continuous monitoring of adherence to inhalation therapies, weekly home spirometry using electronic devices with data transmission to patients and caring physicians combined with a self-management app, video-conferencing and professional telephone coaching. In standard-of-care, scheduled visits with the caring CF physician take place once quarterly

This study is a randomized controlled, multi-centre, non-blinded trial in patients with CF. We present the second version of the study protocol issued 03 December 2020 following the first version issued 24 September 2020. First patient in was in March 2021 and recruitment continues until 30 September 2021. A standard-of-care arm is compared to an intervention arm using the following technologies: objective, continuous monitoring of adherence to inhalation therapies, weekly home spirometry using electronic devices with data transmission to patients and caring CF physicians combined with a self-management app, video-conferencing and professional telephone coaching (see Fig. 1). In standard-of-care, scheduled visits with the caring CF physician take place once quarterly. Further visits only occur at the patient’s request in cases where the patient’s health status deteriorates. In order to evaluate the study interventions in different medical settings, participants will be recruited at CF centres at four sites: (1) two major cities (Charité – Universitätsmedizin Berlin; Cystic Fibrosis Centre Munich-West), (2) one conurbation (University Medicine Essen), and (3) one rural area (University Medicine Rostock and three associated doctor’s practices). The Ethics committee of Charité – Universitätsmedizin Berlin, Germany, has approved the study (application number EA2/241/20) and the study was registered with the German Clinical Trials Register (Identifier: DRKS00024642). PARI Pharma Ltd. provides the eTrack Controller and the 2net Hub (Philips, North America). All study costs are covered by the German Innovation Fund with the funding number: 01NVF19008. The funders had no role in the design, management, analysis and reporting of the study.

### Study population

To be eligible for the study, participants must have a confirmed diagnosis of CF, be age 12 years or older and be able to give written informed consent or assent. In case of minor participants, the written informed consent of the legal guardian must also be provided. Study physicians obtain consent and assent. In addition, participants must have had at least one pulmonary exacerbation in the year before enrolment, a lung function with a forced expiratory volume in one second (FEV1) < 90% of the predicted value at the day of inclusion and if receiving a CFTR modulator therapy, participants must be stable on this treatment for the last three preceding months with no planned change in CFTR modulator treatment during the study period. A pulmonary exacerbation is defined according to the Bilton criteria (modified Fuchs criteria) as an episode of decreased pulmonary function caused by infection and treated with additional antibiotic therapy for at least two of the following reasons (21, 22): change in the amount of sputum or colour, increased cough, malaise, fatigue, lethargy, anorexia or weight loss, decrease in FEV1 by 10 percent or more from the last previously recorded values and/or radiographic changes indicative of pulmonary infection or increased dyspnoea.

Exclusion criteria include an acute depressive or psychotic episode, substantial immobility, no prescribed inhalation therapy, insufficient knowledge of the German language, lack of possession of a smartphone, being post-lung-transplantation, not being able to perform lung function testing, or lung function testing is contraindicated (e.g., because of pneumothorax or lung surgery within the previous three months). Participation in other intervention studies is also an exclusion criterion unless it is an open label study with the participation of already three or more months without planned cessation within our study duration.

### Randomization

A 1:1 randomization to the intervention group (IG) and control group (CG) is performed by an electronic-data-captures-system (EDC-Systems, secuTrial^®^) across the participating centres following study inclusion. Participants are stratified by age (12–17 years and ≥ 18 years of age), sex, CFTR modulator therapy, and lung function (FEV1 < 40% and FEV1 ≥ 40% predicted value at baseline/screening).

### Study flow and study intervention

**Fig. 2:**
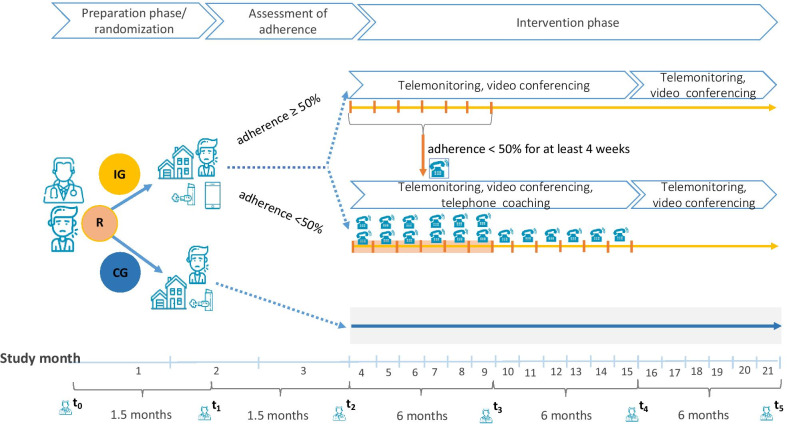
*Study phases and procedures.* During the preparation phase, participants are 1:1 randomized (R) to the intervention group (IG) and control group (CG), study procedures are explained and the correct handling of the study devices is being trained. The IG obtains a telemedicine capable nebulizer (eFlow rapid + nebulizer system, PARI Pharma GmbH, Germany), a home spirometry (mySpiroSense, PARI GmbH, Germany) and a CF therapy management app (PARI Connect App, PARI Pharma GmbH, Germany). The CG also receives a telemedicine capable nebulizer (eTrack Controller with eFlow rapid nebulizer, PARI Pharma Ltd, Germany, and 2net Hub, Philips, North America). During the phase of assessment of adherence, adherence to inhalation therapy is calculated. Adherence data will be available to the patient, caring CF physicians and coach in the IG, but not in the CG. At the end of the assessment phase, the IG is further stratified according to baseline adherence (adherence < 50% group and adherence ≥ 50% group). During the intervention phase, continuous digital monitoring of adherence to inhalation therapies and of lung function is performed in the IG. Participants of the IG can make use of video-conferencing with their caring CF physician up to three times per quarter. Participants of the IG with an adherence < 50% during the assessment phase additionally receive telephone coaching. In the case where mean adherence drops below 50% for at least four weeks in the first six months of the intervention phase in patients with initially good adherence (≥ 50%), these participants will receive a crisis talk followed by telephone coaching. In the last six months of the intervention phase, only monitoring and optional video-conferencing take place.  = quarterly visit outpatient clinic,  = intensive telephone coaching,  telephone coaching,  crisis phone call, t_0_ = inclusion into study, t_1_ = start assessment of adherence, t_2_ = start study intervention, t_3_ = six months of study intervention, t_4_ = 12 months of study intervention, t_5_ = end of study intervention

Study flow and study intervention are depicted in Fig. 2 and an overview of applied survey instruments and collected data is provided in Table 1. During the preparation phase, which lasts up to six weeks, study procedures are explained to the participants, informed consent is obtained, and the participant is trained in the correct handling of the study devices.

**Table 1:** Overview of applied survey instruments and collected data for both the intervention and control group

Survey instrument/collected data	t0	t1	t2	t3	t4	t5
Anthropometric data	IG/CG		IG/CG	IG/CG	IG/CG	IG/CG
Sociodemographic data	IG/CG		IG/CG	IG/CG	IG/CG	IG/CG
Clinical history	IG/CG		IG/CG	IG/CG	IG/CG	IG/CG
Clinical findings	IG/CG		IG/CG	IG/CG	IG/CG	IG/CG
Lung function test	IG/CG		IG/CG	IG/CG	IG/CG	IG/CG
Therapy plan	IG/CG		IG/CG	IG/CG	IG/CG	IG/CG
Pulmonary exacerbations since last study visit	IG/CG		IG/CG	IG/CG	IG/CG	IG/CG
Time period until first pulmonary exacerbation since beginning of study intervention				IG/CG	IG/CG	IG/CG
Time between pulmonary exacerbations since last study visit	IG/CG		IG/CG	IG/CG	IG/CG	IG/CG
CF-associated hospital admissions since last study visit	IG/CG		IG/CG	IG/CG	IG/CG	IG/CG
Days absent from work or school since last study visit				IG/CG	IG/CG	IG/CG
CF-associated medical treatment and care since last study visit				IG/CG	IG/CG	IG/CG
Telemonitoring (App)						
Continuous monitoring of adherence to inhalation therapy		IG/CG	IG/CG	IG/CG	IG/CG	IG/CG
		continuously				
Weekly lung function measured by home spirometry^a^			IC	IC	IC	IC
			at least 1x/week			
Patient questionnaire						
Quality of life: EQ-5D-5L /. EQ-5D-Y-5L	IG/CG		IG/CG	IG/CG	IG/CG	IG/CG
Quality of life: CFQ-R	IG/CG		IG/CG	IG/CG	IG/CG	IG/CG
Beck Depression Inventar	IG/CG		IG/CG	IG/CG	IG/CG	IG/CG
Expectations and their fulfilment regarding the new form of care	IG/ relatives					IG/ relatives
Telephone interview						
acceptance, satisfaction, potential for optimization, barriers and implementation into standard care						IG/physician/ relatives

IG = intervention group, CG = control group, t_0_ = inclusion into study, t_1_ = start assessment of adherence, t_2_ = start study intervention, t_3_ = six months of study intervention, t_4_ = 12 months of study intervention, t_5_ = end of study intervention

^a^Lung function is measured at least weekly by home spirometry

The IG obtains a telemedicine capable nebulizer (eFlow rapid + nebulizer system consisting of an eTrack Controller with eFlow rapid nebulizer handset, PARI Pharma GmbH, Germany), home spirometry (mySpiroSense, PARI GmbH, Germany) and a CF therapy management app (PARI Connect App, PARI Pharma GmbH, Germany) to support self-management, provide adherence monitoring and transfer lung function values. The inhalation device can be used with all standard nebulized therapeutic agents. Data on date, duration, and inhaled agent will be collected and automatically transferred to a web cloud-based telemedicine data server. The CG also receives a telemedicine capable nebulizer (eTrack Controller with eFlow rapid nebulizer, PARI Pharma Ltd, Germany, and 2net Hub, Philips, North America). Data on adherence will be automatically tracked for final evaluation without any data access for participants or caring CF physicians in this group.

The adherence assessment phase follows the preparation phase. Over a minimum of four weeks, adherence to inhalation therapy will be tracked for both groups to obtain a baseline value for adherence before the intervention phase. Adherence is calculated by the ratio of the number of logged nebulization with a duration of more than one minute and the number of prescribed inhalation therapies. Adherence data will be available to the patient, caring CF physicians and coach in the IG, but not in the CG. The IG is further stratified according to baseline adherence (adherence < 50% subgroup and adherence ≥ 50% subgroup).

During the intervention phase of 18 months duration, continuous digital monitoring of adherence to inhalation therapies and of lung function is performed in the IG. Performed inhalation therapies are compared to individual therapy plans and adherence is calculated and displayed both to the patient and caring CF physician. Lung function measurements via home spirometry are performed at least once per week, ideally at the same time of day and following bronchodilator therapy (if applicable). A progressive graph is generated from lung function data and provided to the CF physician. Additionally, the IG participants can make use of video-conferencing with their caring CF physician up to three times per quarter. While the patient can request video-conferencing, the CF physician will also recommend this option if the patient's FEV1 decreases by > 5% compared to the mean of the previous two measurements.

All the IG participants will receive continuous monitoring and optional video-conferencing, and participants with an adherence < 50% during the assessment phase will receive telephone coaching. Psychologist from Thieme TeleCare Ltd. will carry out telephone coaching. It is based on a necessity-concern-framework and consists of two phases: change talk and commitment talk. The coaching's primary goals include supporting the patient with disease management, creating awareness of possible issues with therapy adherence, increasing motivation for adherence, strengthening the perception of risks of non-adherence, and involving parents, partners, or close relatives (23). This coaching will take place twice per month for the first six months and once per month in the following six months. In the case where mean adherence drops below 50% for at least four weeks in the first six months of the intervention phase in patients with initially good adherence (≥ 50%), these participants will receive a crisis talk and intensive telephone coaching (twice/months) for the following six months. This intervention will be followed by a phase of reduced telephone coaching, whereby the number of months with reduced coaching depends on the time left in the intervention phase. In the last six months of the intervention phase, only monitoring and optional video-conferencing occur to assess the effect of the coaching. In the CG, continuous digital monitoring of adherence to inhalation therapies is performed as well, but calculated adherence is neither displayed to the patient nor to the CF physician.

### Measures

Anthropometric and sociodemographic data, information on clinical history, lung function, pulmonary exacerbations (number and time between exacerbations) and CF-associated admissions to hospital, number of days absent from work or school and CF-associated medical treatment and care are collected at pre-determined time points during the study period. Lung function measurement is performed at each study visit using the SpiroSense Pro^®^ spirometer (PARI GmbH, Germany), using the same technology as the home spirometer. Additionally, two different questionnaires, the German CF-questionnaire revised (CFQ-R) in its age-appropriate version and the EQ-5D-5L or EQ-5D-Y-5L in adolescents, respectively, are performed to assess the quality of life of all participants initially, during, and at the end of the study period (24–27). Participants will also be regularly screened for depressive disorders using the Beck Depression Inventar Fast Screen at each study visit (28, 29). Expectations regarding the new form of care applied in this study and their fulfilment are assessed at baseline and the end of the study period (see additional file “Questionnaire expectations conneCT CF”). Participants, their relatives, and CF physicians will be surveyed on acceptance, satisfaction, potential for optimization, barriers and implementation of this new form of care into standard care at the end of the study via a telephone interview (see Table 1).

### Endpoints

The primary endpoint is the time to first protocol-defined pulmonary exacerbation after initiation of the intervention phase. Secondary outcome measures include the number of pulmonary exacerbations, time between pulmonary exacerbations, adherence to inhalation therapy, changes of FEV1 and forced vital capacity (FVC) from baseline, the number of CF-associated hospital admissions, and changes in health-related quality of life (assessed with CFQ-R and EQ-5D-5L). Furthermore, sociodemographic and anthropometric data, number of days absent from work or school, and CF associated medical treatment and care are assessed by explorative analysis, and health care-related costs for both study groups are evaluated.

### Data management

At each study site, research personnel enter data from source documents into the electronic case report form (eCRF, EDC-Systems, secuTrial^®^). Data collected from the PARI Connect App can be accessed via a web-based dashboard. The data from the dashboard and all further data transfer within the study will be performed in pseudonymized form as comma-separated values (CSV) file via secure file transfer protocol (SFTP) server with exception of personal data needed for video-conferencing and telephone coaching. For video-conferencing, a secured web portal (m.Doc GmbH) is established with restricted access only for the participant and study personnel. All study collaborators will have equally access to the final trial dataset. Trial results will be published in peer-reviewed journals. Authorship eligibility is based on substantial contributions to the conception or design of the work; or the acquisition, analysis, or interpretation of data for the work.

### Quality assurance

Quality of medical data is ensured by intensive training of the participants in the use of the nebulizer, the app, and the performance on the home spirometry. Treatment plans entered into the app by the participants will be compared to those entered into the eCRF by the study personnel and checked at each study visit. To ensure safe data collection and data transfer, an extensive data protection concept compliant with GDPR (General Data Protection Regulation) has been elaborated. The study protocol has been designed according the standard ethical and scientific principles such as the declaration of Helsinki, guidelines of CIOMS (Council for International Organization of Medical Sciences) in collaboration with WHO (World Health Organization). Study sites report recruitment progress to all trial collaborators on a monthly basis. In case no data is tracked from the inhalation device or home spirometry, an alert to the study centres is created to ensure adherence to study measures. Study interventions are discontinued on patient’s request or in case of insufficient patient’s adherence to study interventions and study visits. Participant’s data will be collected and evaluated until discontinuation of study participation. An interim analysis will be performed after 12 months following study initiating. All study collaborators will have access to these interim results. Because of the minimal risk of the intervention, possible adverse events will be collected at routine study visits. For the same reason, a formal data monitoring committee is not needed.

### Sample size calculation

Sample size calculation was performed by Private Institute for Applied Health Services Research (inav) GmbH and is based on the following assumptions: (1) primary endpoint: time to pulmonary exacerbation (30), (2) median time to exacerbation is 30% less in the CG compared to the IG based on estimation of involved CF experts, (3) event-free survival within 12 months in 25% of participants (18), (4) a significance level α = 0.05 and power of 1 – β = 0.8, (5) dropout rate of 20% (18) and (6) exponential distribution of survival and proportional hazards. Following these assumptions, a sample size of 391 participants is needed. To account for possible deviations from the assumed survival and dropout rate, we aim to recruit 402 patients. Participants are recruited as part of their routine visit at their CF centre by research personnel.

### Statistical analysis plan

All data will be analysed between IG and CG (cross-sectional analysis) as well as longitudinally. For the descriptive data analysis, adequate location and dispersion measures will be determined, and correlation analysis will be performed. An inferential data analysis will follow this. Depending on the kind of data and data distribution appropriate statistical tests will be applied: e.g. t-test, Wilcoxon-Rank-test, Friedman-tests or variance analysis with repetitive measures in order to identify longitudinal differences; e.g. log rank-test, WiIcoxon–Mann–Whitney-test and Chi-square-test for cross-sectional analysis. To investigate the relationships between the variables, multivariate analysis and regression analysis will be performed. An intention-to-treat-analysis will be used and further evaluated for its sensitivity according to per-protocol-principles and as-treated principles.

For the cost- effectiveness analysis the defined primary outcome parameter will be set against determined costs:

$${\text{ICER}} = \frac{{{\text{Mean}}\;{\text{health}}\;{\text{costs}}\;{\text{in}}\; {\text{IG}} - {\text{Mean}}\;{\text{health}}\;{\text{costs}}\; {\text{in}}\;{\text{ CG}}}}{{{\text{Median}}\;{\text{time}}\;{\text{to}}\;{\text{exacerbation}}\;{\text{ in}}\;{\text{IG}} - {\text{Median}}\;{\text{time}}\;{\text{to }}\;{\text{exacerbation}}\;{\text{in}}\;{\text{CG}} }}$$

To account for uncertainty in estimation of ICER (incremental cost-effectiveness ratio), a non-parametric bootstrapping will be performed. Additionally, cost-effectiveness-acceptability-curves will be generated.

Qualitative data acquired from the telephone interviews will be transcribed verbatim and analysed via the data analysis program MAXQDA.

Using the EDC systems minimizes missing data. In the case missing or implausible data, study centres will be contacted and data possibly corrected. Alternatively, we will assess if appropriate imputation methods can be applied.

## Discussion

The conneCT CF trial is the first large multi-centre randomized controlled trial aiming to evaluate telemedicine’s effect including telemonitoring, self-management app, video-conferencing and telephone coaching on adherence, pulmonary exacerbations, and quality of life in patients with CF.

Digital technologies are widely used among the general population and their importance for health care is being more and more recognized. This development has led to the publication of the first global strategy for digital health by the WHO (31). The appeal of digital technologies in CF care is improved monitoring of therapy and health status by real-time applications leading to improved self-management, the opportunity of earlier intervention in case of lung function decline, better access and reduced travel to specialized centres and reduced risk of cross-infection at clinic visits.

Treatment adherence requires self-management skills, which are also the critical component to successfully managing chronic disease (32). In adolescents, an age group where adherence is particularly challenging, home monitoring was shown to be a feasible adherence intervention and a strong adherence motivator (33, 34). Recently developed data-tracking nebulizers enable patients and physicians to monitor adherence to a variety of inhalation therapies objectively. In the presented trial, information on lung function and adherence to different inhalation therapies can be made easily available on the patient’s smartphone and to the caring CF physician. Additional features of the app such as schedules and patient reminders can help participants build good self-care habits. Providing adherence feedback might also improve the quality of clinical consultations by enabling an open and honest discussion about adherence issues (35). Data tracking nebulizers form the basis of further ongoing trials evaluating different telehealth interventions aiming to increase adherence to nebulizer therapy (Tele-Coaching Intervention to Improve Treatment Adherence in Cystic Fibrosis, ClinicalTrials.gov Identifier: NCT03921229) (36–39). The Tele-Coaching Intervention-trial is a prospective, multi-centre pilot study including 100 patients with CF. An eTrack nebulizer and a vest monitor photo capture are used as measures of adherence. Primary outcome measures focus on patient attrition, intervention acceptability and feasibility and second outcome measures on adherence. The most significant ongoing trial on telemetric measurement of adherence is the CFHealthHub Data Observatory aiming to include as many as possible of the 6,000 adult patients with CF at 20 CF centres in the United Kingdom following a pilot phase and a randomized controlled trial with 608 patients at 19 CF centres (38). Similar to our study, adherence data on inhalation therapy is uploaded and made available on the patients’ smartphone and to the caring CF physician via a healthcare software (CFHealthHub software system). In contrast to our study, this study’s behavioural change intervention is not combined with a personal coaching. The phone-coaching interviews in our study will offer the possibility to address individual barriers for adherence and support patients in developing disease management strategies by psychologists, in addition to the examination and visit at the CF centre. Furthermore, our study is the first study combining adherence interventions with continuous monitoring of lung function and health status. This innovative approach allows simultaneous monitoring of the adherence-modifying interventions and their effectiveness. The benefit of home monitoring on early detection of pulmonary exacerbations, lung function and well-being were shown in several studies (17, 20, 40, 41).

Nevertheless, a large randomized controlled trial on home-monitoring in patients with CF, the early intervention in cystic fibrosis exacerbation (eICE) trial, resulted in the detection of more exacerbations than with standard care. However, despite the treatment of these events, this intervention did not improve lung function (18). Tight monitoring of patients’ health status and lung function in the presented study might also lead to the paradoxical effect of increasing numbers of pulmonary exacerbations in the IG compared to the CG. Early alert of the caring physician to decreasing lung function and facilitated access to medical care by video-conferencing aim to adjust treatment in a timely manner and prevent disease progression in the long-term. A 52-week study period, as used in the eICE trial, might have been too short to evaluate the effect of home monitoring, especially in relatively stable patients with infrequent pulmonary exacerbations. In our trial, we therefore chose a more extended intervention phase of 18 months.

The eICE trial highlighted the issue of adherence to telemedicine: adherence with the once weekly transmission of home spirometry data was 50%, with twice-weekly data transmission only 19%. Data transmission issues were also reported as a limiting factor in previous telemedicine studies (40, 42). In the presented study, adherence to telemedicine is facilitated by reminders set in the smartphone app and automatic data transfer from the spirometer and inhalation device without any action by the participant, except using the app and accepting the data transfer.

However, home monitoring bears the risk of adverse effects including increased anxiety caused by increased awareness of deterioration in lung function (43). In the presented study, the possibility to request video-conferencing in addition to quarterly visits addresses this issue and provides an opportunity for additional, uncomplicated contact with the caring CF physician. The CF physician can also offer video-conferencing in case home monitoring data show deterioration in lung function facilitating intensified care compared to the CG. In addition, patients are screened for quality of life and depression regularly, and a psychologist is consulted if the screening indicates an increased psychological burden.

In telemedicine studies relying on a particular technical infrastructure, concerns regarding generalising of the results might be raised. These concerns are addressed by enrolling patients from different geographical regions and different treatment facilities (university hospital, outpatient clinic and doctor's practice). Another concern focuses on the quality of the data collected remotely during clinical trials (44). Staff and participants will be extensively trained in the handling of the devices. Lung function will be measured at each study visit using a spirometer (Spiro Sense Pro, PARI Ltd., Germany) that uses the same technology as the home spirometer, serving as further quality control of the lung function measurement at home.

Recently, the new triple CFTR modulator therapy containing the correctors tezacaftor and elexacaftor combined with the potentiator ivacaftor received approval for patients aged 12 years and older who have at least one F508del mutation by the U.S. Food and Drug Administration (FDA) and for patients who are homozygous or compound heterozygous for the F508del mutation in combination with a minimal function mutation by the European Medical Agency (EMA) (45, 46). In light of these highly effective CFTR-directed therapies, the role of inhalation therapies in CF treatment is to be defined and objective data on treatment adherence and lung function will inform these discussions.

New forms of health care are to be evaluated for their impact on patients’ health and also for their cost-effectiveness compared to traditional care. Data on the cost-effectiveness of telemedicine in CF care are scarce, but two studies from Italy evaluating telemedicine estimated cost savings per patient of around 5,000€ annually and 40,000€ over a 10-year-period (47, 48). Nevertheless, conclusions drawn from one health system might not be transferable to a different health system and more data from different settings are needed before a more general statement on the cost-effectiveness of telemedicine can be made.

Our study has some limitations: due to the study design’s nature, blinding of the patients and staff is not possible. Participation in this trial will be offered to all patients meeting the inclusion criteria. Nevertheless, only very motivated patients may agree to participate, leading to a selection bias. Additionally, a performance bias could occur because staff and patients of the participating centres might be more aware of the issue of adherence. This bias might lead to an increase in adherence also in the CG possibly diminishing our ability to detect differences in adherence between the IG and CG.

The coronavirus disease 2019 (COVID-19) pandemic has highlighted the importance and potential of digital technologies in patient care, and different strategies have been rapidly implemented (49–51). Scientific evidence for the feasibility, clinical benefit and cost-effectiveness of these interventions in patients with chronic conditions like CF is urgently needed to form the basis of a regulatory framework to authorize, integrate, and finance telemedicine services (52).

In summary, this study offers the opportunity to establish structures for secure data transmission, evaluate the use of telemedicine capable devices and adherence interventions on lung health and its cost-effectiveness, and possibly pave the way for implementation of telemedicine in routine care for patients with CF.

## Supplementary Information

**Additional file 1:** Title of data: Questionnaire expectations conneCT CF. Description of data: English translation of questionnaire on expectations of the program conneCT CF and their fulfilment at the beginning and the end of the study.

## Data Availability

Data sharing is not applicable to this article as no datasets were generated or analyzed during the current study.

## Abbreviations

CF: Cystic fibrosis
CFQ-R: Cystic fibrosis-questionnaire revised (German version)
CFTR: Cystic fibrosis transmembrane conductance regulator
CG: Control group
CIOMS: Council for International Organization of Medical Sciences
CSV: Comma-separated values
eCRF: Electronic case report form
eICE: Early intervention in cystic fibrosis exacerbation
EMA: European medical agency
FDA: Food and Drug Administration
FEV1: Forced expiratory volume in 1 s
FVC: Forced vital capacity
GDPR: General data protection regulation
ICER: Incremental cost-effectiveness ratio
IG: Intervention group
MPR: Medication possession ratio
SFTP: Secure file transfer protocol
U.S.: United States
WHO: World Health Organization
