# Supplementary material for: A multi-centre, randomized, controlled trial on coaching and telemonitoring in patients with cystic fibrosis: conneCT CF
Source: BMC Pulm Med. 2021 Apr 21;21:131. doi: 10.1186/s12890-021-01500-y (PMC8058751; doi:10.1186/s12890-021-01500-y)
Supplement: Supplementary file 1 — Additional file 1: Title of data: Questionnaire expectations conneCT CF. Description of data: English translation of questionnaire on expectations of the program conneCT CF and their fulfilment at the beginning and the end of the study. [file 12890_2021_1500_MOESM1_ESM.docx]

Questionnaire on expectations of the program conneCT CF

Questionnaire at t0

| Who is filling out the questionnaire | Patient  Relatives/Parents |
| --- | --- |
| What advantages do you expect from participation in this program? |  |
| Do you think the program improves your adherence to medication? | Yes  No  If yes, please specify |
| Do you think the program can lead to improvement of your symptoms? | Yes  No  If yes, please specify |
| In your opinion, what kind of negative effects could this program have? |  |

Questionnaire at t5

| Who is filling out the questionnaire | Patient  Relatives/Parents |
| --- | --- |
| What advantages did you have from participation in this program? |  |
| Do you think the program has improved your adherence to medication? | Yes  No  If yes, please specify |
| Do you think the program has lead to improvement of your symptoms? | Yes  No  If yes, please specify |
| Did any negative effects due to this program occur? | Yes  No  If yes, please specify |
